# A comprehensive dataset and image-set for exploring buccal dental microwear in late prehistory farming groups from northeastern Iberian Peninsula

**DOI:** 10.1016/j.dib.2024.110929

**Published:** 2024-09-10

**Authors:** Raquel Hernando

**Affiliations:** a Centro Nacional de Investigación sobre la Evolución Humana (CENIEH), Paseo Sierra de Atapuerca 3, 09002 Burgos, Spain; b Catalan Institute of Human Paleoecology and Social Evolution (IPHES-CERCA), Edificio W3, Campus Sescelades URV, Zona Educacional, 4, 43007 Tarragona, Spain; c Departament d'Història i Història de l'Art, Universitat Rovira i Virgili, Tarragona, Spain

**Keywords:** Paleoantropology, Paleodiet, Prehistory, Teeth, Holocene

## Abstract

This data article presents a comprehensive buccal dental microwear raw database, accompanied by all corresponding archaeological sample micrographs acquired through a ZEISS Axioscope A1 optical microscopy (OM). The dataset includes teeth specimens from 88 adult individuals, representing eight distinct groups spanning the Middle-Late Neolithic to the Middle Bronze Age from the northeastern Iberian Peninsula. These groups include Cova de l'Avi, Cova de Can Sadurní, Cova de la Guineu, Cova Foradada, Cova del Trader, Roc de les Orenetes, Cova del Gegant, and Cova dels Galls Carboners.

The data collection process was based on the use of optical microscopy to obtain dental microwear patterns, with a specific focus on the buccal surface of the teeth. To facilitate future comparative studies, we have also included all the micrographs obtained with the optical microscopy and the processed images with the counted striations. The presentation of this extensive dataset sets a base for future research on dental microwear patterns and dietary variations across various prehistoric periods.

Specifications Table

**Table:** 

Subject	Archaeology
Specific subject area	Human teeth: analysis of buccal dental microwear though optical microscope
Data format	Raw, Analyzed and Filtered
Type of data	Table, Image
Data collection	The dental remains were observed with a ZEISS Axioscope A1 optical microscope, following the methodology outlined by [1]. After image acquisition, the images were cropped to an area of 0.56 mm^2^. Following the established methodology with the Scanning Electron Microscope [2], the total number of striations (TN), the average striation length (XT, in µm), the width of buccal striations (W), and each striation's orientation were semi-automatically recorded using the open-access software, ImageJ®. The analysis focused exclusively on teeth with well-preserved enamel, excluding those displaying evidence of post-mortem damage.
Data source location	• Institution: Catalan Institute of Human Paleoecology and Social Evolution (IPHES) • City/Town/Region: Tarragona • Country: Spain • Latitude and longitude (and GPS coordinates, if possible) for collected samples/data: Cova de l´Avi (Vallirana, Barcelona) [41°22′47.8″N 1°53′38.7″E] Cova Can Sadurní (Begues, Barcelona) [41°20′43.4″N 1°54′43.9″E] Cova de la Guineu (Font-Rubí, Barcelona) [41°26′32.6″N 1°34′31.4″E] Cova Foradada (Calafell, Tarragona) [41°12′17.8″N 1°34′51.5″E] Cova del Trader (Cubelles, Barcelona) [41°13′56.4″N 1°39′12.9″E] Roc de les Orenetes (Queralbs, Girona) [42°23′30.6″N 2°09′45.6″E] Cova dels Galls Carboners (Mont-ral, Tarragona) [41°17′16.9″N 1°05′20.9″E] Cova del Gegant (Sitges, Barcelona) [41°13′25″N 1°46′27″E]
Data accessibility	Repository name: Zenodo https://zenodo.org/ Data identification number: 10.5281/zenodo.7267735 Direct URL to data: https://zenodo.org/doi/10.5281/zenodo.7267735
Related research article	Hernando, R; Moreno-Ibáñez, M.Á., Carbonell, E., Cebrià, A., Daura, J., Díez-Canseco, C., Edo, M., Fullola, J., Morales, J.I., Oms, F.X., Ramírez-Pedraza, I., Sanz, M., Subirà, M.E., Tornero, C., Vergès, J.M., Lozano, M. (2024). Eating through time: Understanding dietary practices across late Prehistory in the northeastern Iberian Peninsula. Am. J. Biol. Anthropol. e24950. DOI: 10.1002/ajpa.24950

## 1 Value of the Data

• These data offer a unique perspective on dietary variations during the Late Prehistory of the northeastern Iberian Peninsula, providing valuable information through the examination of buccal dental microwear patterns.
• The raw data stored in this repository serve as an extensive resource, offering researchers a comprehensive dataset for in-depth exploration into dental microwear studies and Late Prehistory in the northeastern Iberian Peninsula.
• By making this data publicly available, we aim to promote transparency in dental microwear methodologies. This initiative establishes a solid base for future research into dietary practices across different periods. This dataset not only serves as a valuable tool for researchers specializing in dental microwear for comparative purposes but also contributes to the broader archaeology scientific community by providing a source of raw data for diverse analytical approaches and interpretations. Additionally, this dataset holds significant potential for prospective students who are interested in exploring this field. Furthermore, the inclusion of all raw images obtained through OM and after processing, ensures the repeatability and reproducibility of this work, further enhancing its scientific reliability.

## 2 Background

This dataset seeks to contribute to the current understanding of dietary variations in the Late Prehistory of the northeastern Iberian Peninsula, a period of significant dynamism and complexity, by examining buccal dental microwear patterns [3]. This technique has emerged as a valuable tool for understanding the physical and mechanical properties of consumed foods, providing a crucial line of evidence for the study of dietary practices (e.g., [2,[4], [5], [6]]). For that reason, this data contributes valuable raw data to further the understanding of how dietary dynamics influenced various subsistence strategies during Late Prehistory in the northeastern Iberian Peninsula.

In addition to our research focus, another driving force behind this project is the lack of a solid reference framework when conducting studies on dental microwear in humans. To address this, we have not only chosen to share the dataset openly but also all the processed images. This initiative aims to provide other researchers with an important resource for meaningful comparisons in the future.

## 3 Data Description

**Fig. 1:**
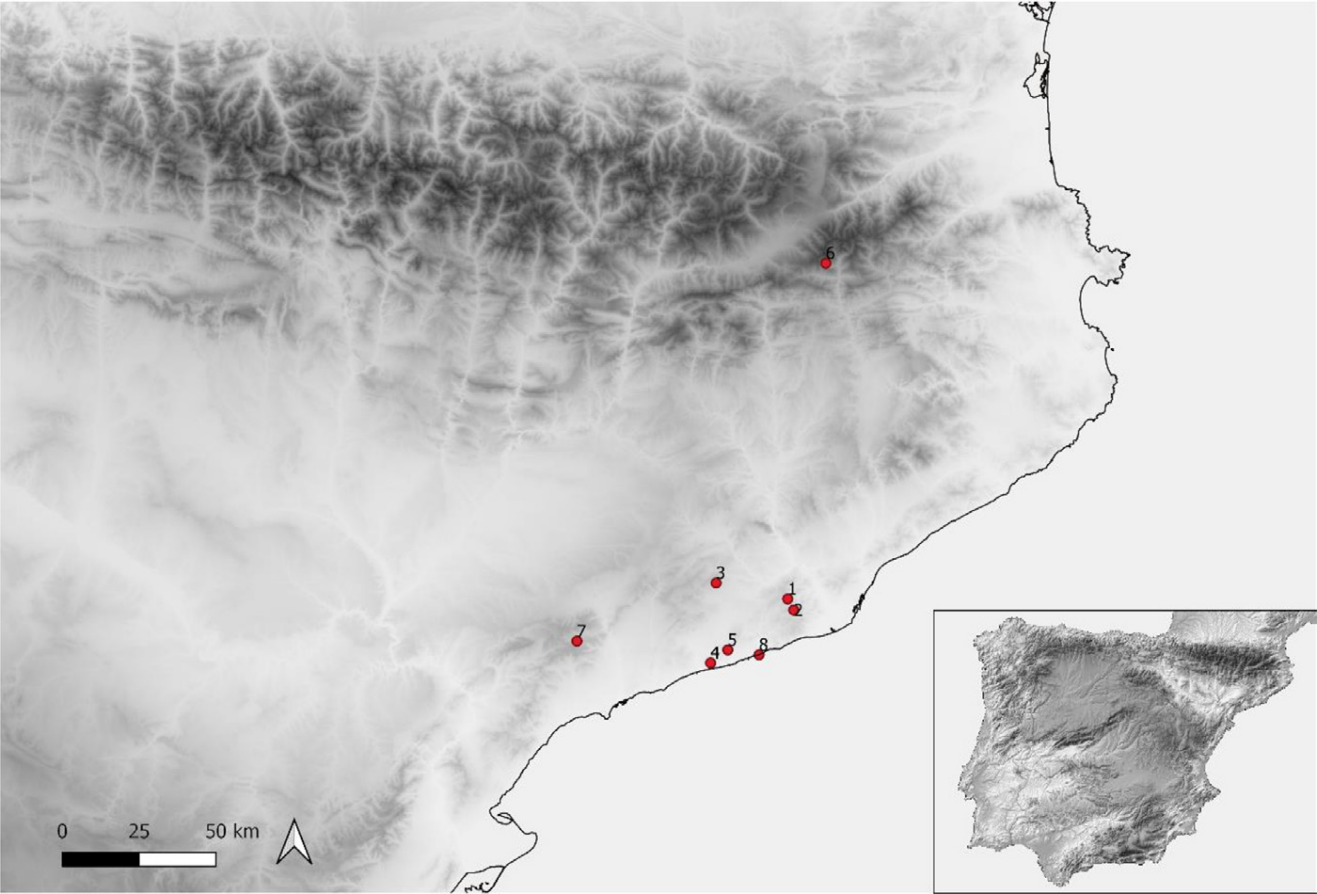
Map showing the location of the sites employed in this study. Archaeological sites: 1. Cova de l'Avi; 2. Cova de Can Sadurní; 3. Cova de la Guineu; 4. Cova Foradada; 5. Cova del Trader; 6. Roc de les Orenetes; 7. Cova dels Galls Carboners; 8. Cova del Gegant.

A map of the northeastern region of the Iberian Peninsula shows the locations of sites included in this study (Fig. 1; Table 1).

**Table 1:** Archaeological sites of the samples analyzed with their chronologies.

Site	Chronology	References
Cova de l´Avi	Middle-Late Neolithic	[7]
Cova de Can Sadurní	Late Neolithic-Chalcolithic	[8]
Cova de la Guineu	Late Neolithic-Chalcolithic	[9]
Cova Foradada	Late Neolithic-Chalcolithic	[10]
Cova del Trader	Late Neolithic-Chalcolithic-Bronze Age	[11]
Roc de les Orenetes	Late Chalcolithic-Middle Bronze Age	[12]
Cova del Gegant	Middle Bronze Age	[13]
Cova dels Galls Carboners	Middle Bronze Age	[14]

### 3.1 Dataset structure

The dataset is organized based on individual identifiers (ID) for the buccal surface, categorized into different groups: (CA: Cova de l´Avi, CS: Can Sadurní, GN: Cova de la Guineu, FO: Cova Foradada, RO: Roc de les Orenetes, CG: Cova del Gegant, GC: Galls Carboners.). Sex abbreviations: female (F), male (M), undetermined (U). Abbreviations for tooth types correspond to jaw (upper or lower: U or L), side (left or right: L or R), and position in tooth row (first or second: 1 or 2) for molars (M) or premolars (P3 or P4). The dental microwear variables are TN: total number of striations; XT: length of the striations in µm; sd: standard deviation; the orientation indexes (vertical and horizontal); and the width of the striations in µm (W). Variables depend on the orientation (MD: mesio-distal; V: vertical; H: horizontal; DM: disto-mesial).

### 3.2 Micrograph set folders

The micrographs are grouped in folders by archaeological sites. Within each folder there are the micrographs of each tooth in their raw form. Inside each of these site folders there is a subfolder containing the processed micrographs with the striations counted.

The nomenclature used for each micrograph consists of: Site-Year-ID-Tooth-Surface-Photo Number, corresponding to the nomenclature used in the database.

## 4 Experimental Design, Materials and Methods

The dental microwear analysis were conducted using both original teeth and high-resolution replicas. Direct observation of the original specimens was prioritized whenever possible; however, in specific instances (e.g., when teeth were *in situ* within the jaws), high-resolution replicas were generated to facilitate improved manipulation under the microscope.

### 4.1 Cleaning and replica procedures

The replica procedure consists of three consecutive steps: First, we cleaned of the original teeth with a cotton swab soaked in 99 % acetone to remove any adhering sand, dust, or adhesive residue. Subsequently, the mold or negative impression was made with dental silicone (Colténe President Plus Jet regular body). Two impressions were taken of each tooth. Finally, the second impression was selected to produce the high-resolution replica using Epo-Tek 301, Epoxy Technology, Inc. One of the advantages of this method is that it is not necessary to coat the epoxy replica before inspection under the optical microscope. The replica can be observed under the microscope either with or without the coating, offering flexibility in the analysis.

### 4.2 Buccal dental microwear analysis using the optical microscope (OM)

A ZEISS Axioscope A1 optical microscope equipped with a Differential Interference Contrast system (DIC) and a Nomarski prism was used. Two Epiplan lenses were used: a 5×/0.13 and a 10×/0.20. The camera used with the OM is a 6.3 MP Color Blackfly S USB 3.0 Camera, 1/1.8″, FLIR, and the software used to control image acquisition is Kivy Capture MIC Z.

Tooth orientation was standardized by keeping the occlusal side at the top. Micrographs were taken at 100× magnifications (10x objective plus 10x oculars) on the medial third of the buccal surfaces with a horizontal field of view of 1464 µm [1].

The resulting micrographs were processed with the open software Helicon Focus 5.3 image processing program, using the pyramidal C-mount method, which performs focus stacking.

### 4.3 Image processing and semi-automatic variable acquisition

After the image acquisition, the micrographs were cropped to cover an area of 0.56 mm^2^ [2]

**Fig. 2:**
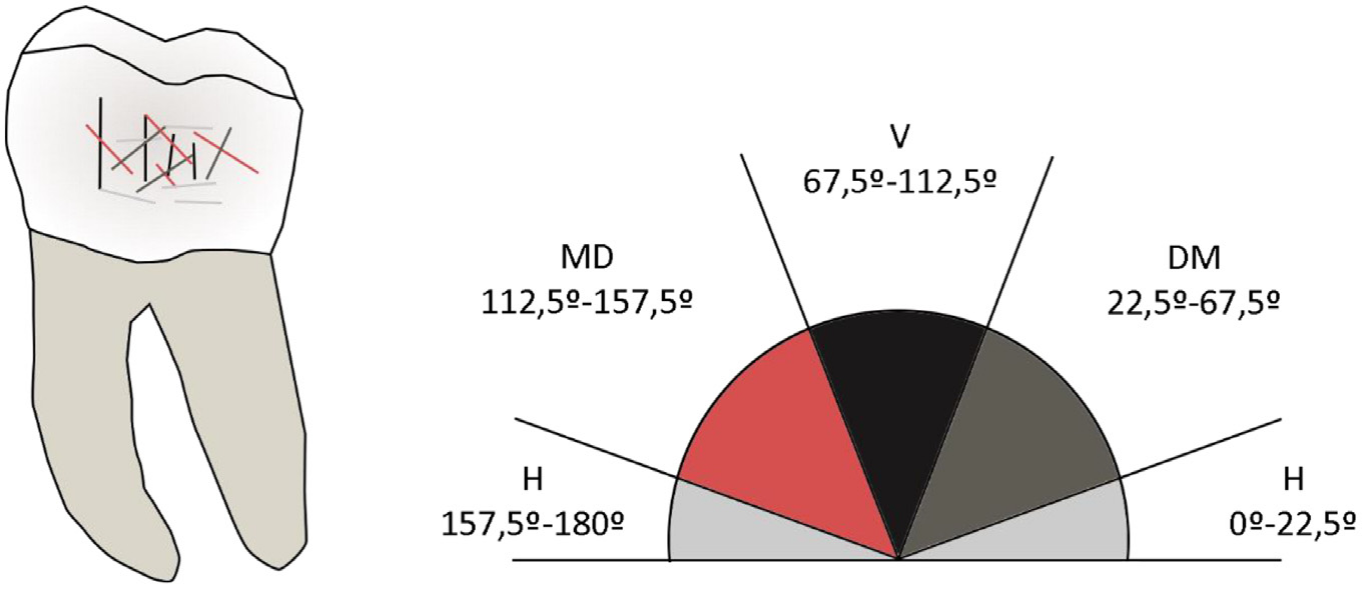
Scheme of the distribution of the striations on the buccal surface of the tooth depending on their orientation, categorized in 45° angles: horizontal (H), mesiodistal (MD), vertical (V) and distomesial (DM). Image modified from [2].

Then, the total number of striations in the buccal surface (BTN), the average length (XT, in µm), and their orientation (0°–180°, divided into 45° intervals according to their orientation (Fig. 2): horizontal (H) (0°–22.5°; 157.5°–180°), vertical (V) (67.5°–112.5°), mesiodistal (MD) (Lower Left Molar/Upper Right Molar [LL/UR]: 112.5°–157.5°; Upper Left Molar/Lower Right Molar [UL/LR]: 22.5°–67.5°); distomesial (DM) (Lower Left Molar/Upper Right Molar [LL/UR]: 22.5°–67.5°; Upper Left Molar/Lower Right Molar [UL/LR]: 112.5°–157.5°) were computed using open-source image processing software, Image J (version 1.52p), (https:// imagej. nih. gov/ ij/ index. html), following the established methodology with the Scanning Electron Microscope [2,15], Two orientation indexes were calculated: total horizontal/total number (NH/BTN) and total vertical/total number (NV/BTN) [5]. In addition, we explored the variable maximum width of buccal striations (W). To determine the maximum width of buccal striations, we measured the mean of the five widest striations in micrometers (µm) [3].

## Limitations

None

## Ethics Statement

All research was conducted using bioarchaeological skeletal materials. These collections are currently curated by the Catalan Institute of Human Paleoecology and Social Evolution. No data were collected from modern human populations or individuals with known relatives.

## CRediT Author Statement

Raquel Hernando: Conceptualization, Data curation, Methodology, Formal analysis, Writing-Original and draft, Review and Editing.

## Data Availability

Buccal dental microwear micrographs from different Late Prehistory farming groups obtained with optical microscopy (Original data) (ZENODO).
